# Supplementary material for: A high-resolution mRNA expression time course of embryonic development in zebrafish
Source: eLife. 2017 Nov 16;6:e30860. doi: 10.7554/eLife.30860 (PMC5690287; doi:10.7554/eLife.30860)
Supplement: Supplementary file 6. [file elife-30860-supp6.zip › biolayout-clusters-files/Cluster247-genes.html]

Cluster247


# Cluster247: Genes

| Ensembl ID | Gene Name | Chr | Start | End | Biotype |
| --- | --- | --- | --- | --- | --- |
| ENSDARG00000105000 | C2CD2 | 10 | 33361919 | 33399320 | protein\_coding |
| ENSDARG00000013397 | bnip2 | 25 | 33761198 | 33783012 | protein\_coding |
| ENSDARG00000034823 | copg2 | 4 | 15984786 | 15997436 | protein\_coding |
| ENSDARG00000013346 | fam208ab | 23 | 19547592 | 19560355 | protein\_coding |
| ENSDARG00000013776 | thop1 | 11 | 7173186 | 7202516 | protein\_coding |
| ENSDARG00000055930 | zc3h7b | 12 | 19036958 | 19063991 | protein\_coding |
